# Vitamin D’s Effect on Immune Function

**DOI:** 10.3390/nu12051248

**Published:** 2020-04-28

**Authors:** Pieter-Jan Martens, Conny Gysemans, Annemieke Verstuyf, Chantal Mathieu

**Affiliations:** Clinical and Experimental Endocrinology (CEE), KU Leuven, Campus Gasthuisberg O&N1, Herestraat 49, box 902, 3000 Leuven, Belgium; pieterjan.martens@kuleuven.be (P.-J.M.); conny.gysemans@kuleuven.be (C.G.); mieke.verstuyf@kuleuven.be (A.V.)

**Keywords:** vitamin D, 1,25-(OH)2D3, immune system, autoimmune disease, infectious disease, type 1 diabetes, multiple sclerosis, rheumatoid arthritis

## Abstract

Ever since its discovery by Windhaus, the importance of the active metabolite of vitamin D (1,25-dihydroxyvitamin D_3_; 1,25-(OH)2D_3_) has been ever expanding. In this review, the attention is shifted towards the importance of the extra-skeletal effects of vitamin D, with special emphasis on the immune system. The first hint of the significant role of vitamin D on the immune system was made by the discovery of the presence of the vitamin D receptor on almost all cells of the immune system. In vitro, the overwhelming effect of supra-physiological doses of vitamin D on the individual components of the immune system is very clear. Despite these promising pre-clinical results, the translation of the in vitro observations to solid clinical effects has mostly failed. Nevertheless, the evidence of a link between vitamin D deficiency and adverse outcomes is overwhelming and clearly points towards avoidance of vitamin D deficiency especially in early life.

## 1. Introduction

The significance of vitamin D for health was first demonstrated by the discovery that its deficiency causes rickets in children and osteomalacia in adults [1]. The identification of vitamin D was groundbreaking, and Windaus was awarded the Nobel Prize in 1938 for this discovery. Despite its name, it is not stricto sensu a vitamin, but in fact, a prohormone, as humans are not exclusively dependent on it by their diet. Vitamin D can be obtained from ultraviolet (UV)B-dependent (wavelength 290–315 nm) endogenous production, from the diet and from supplements [2]. The two major forms of vitamin D (calciferol) are vitamin D_2_ (ergocalciferol) and vitamin D_3_ (cholecalciferol) [3]. Vitamin D_2_ is formed by UV irradiation of ergosterol from vegetable origins, such as yeast and mushrooms, making them naturally rich sources of vitamin D. Vitamin D_3_ is formed in the skin by the UV irradiation of 7-dehydrocholesterol [2]. As only 1,25-dihydroxyvitamin D_3_ (1,25-(OH)_2_D_3_ or calcitriol) is the active vitamin, both 25- and 1α-hydroxylation are required for activation. The 25-hydroxylation happens in the liver by at least five enzymes (i.e., CYP2DII, CYP2D25, CYP3A4, CYP2R1, and CYP27A1). The serum 25-hydroxyvitamin D_3_ (25-(OH)D_3_) reflects the nutritional vitamin D status. The next step to obtain active vitamin D is 1α-hydroxylation (CYP27B1). This enzyme is expressed by many cell types (i.e., skin, immune cells, bone cells, placenta), but is present at the highest concentration in the kidney proximal tubule cells. The activity of the 1α-hydroxylase enzyme in the kidney is highly regulated by calcium and phosphate. Regulation of the 1α-hydroxylase enzyme in the other tissues has only little feedback inhibition. Breakdown of both 25-(OH)D_3_ and 1,25-(OH)_2_D_3_ is done by the same 24-hydroxylation enzyme (CYP24A1). Vitamin D and its metabolites in the circulation are bound to the multifunctional vitamin D-binding protein (DBP), which, besides the transport of vitamin D, also functions as modulator of inflammatory and immune responses as well as a regulator of bone development [1,2,3,4].

The classic vitamin D receptor (VDR) belongs to the nuclear receptor superfamily. Ligand binding results in heterodimerization with the retinoic X receptor (RXR). This complex then classically binds to the vitamin D responsive elements (VDRE) in the promotor region of target genes to exert the genomic effects of 1,25-(OH)_2_D_3_ [5]. Non-genomic effects of calcitriol proceed via the binding of 1,25-(OH)_2_D_3_ to a membrane bound VDR complexed to caveolin-1 [6]. The pleiotropy of vitamin D effects is ever expanding, and vitamin D analogs are produced to help in this exploration [7]. 

To express clinically relevant reserves of vitamin D, serum 25-(OH)D_3_ levels are used (defined as the sum of 25-(OH)D_2_ and 25-(OH)D_3_), as 1,25-(OH)_2_D_3_ is homeostatic regulated and has a short half-life (4–8 h). Nonetheless, the major problem remains the standardization of tests [8]. There are mainly two different methodologies: competitive immunoassays (such as competitive binding-protein assays or radioimmunoassays) and methods based on high-performance liquid chromatography (HPLC) and direct detection with liquid chromatography tandem-mass spectrometry (LC-MS/MS), with the latter methods being the gold standard [9,10,11].

Besides there being issues with standardization of testing, there is also controversy about levels of adequacy. The Endocrine Society defines deficiency as 25-(OH)D_3_ levels less than 20 ng/mL (50 nmol/L) and insufficiency as levels 21–29 ng/mL (52 to 72 nmol/L) [12]. These cut-offs are determined based on parathyroid hormone (PTH) levels and intestinal calcium transporter activity that normalizes as 25-(OH)D_3_ levels reach the current cut-offs [13,14,15]. The Institute of Medicine (IOM) on the other hand, states there is no increased benefit of serum 25-(OH)D_3_ levels above 20 ng/mL (50 nmol/L) and defines deficiency as 25-(OH)D_3_ levels less than 12 ng/mL (30 nmol/L) and insufficiency as levels 12–20 ng/mL (30–50 nmol/L) [16]. Vitamin D intoxication is observed at concentrations higher than 150 ng/mL (374 nmol/L) [2].

Based on these cut-offs, there is a high prevalence (up to 40% in adults) of vitamin D insufficiency in both children and adults. A study in 6275 American children and adolescents aged 1–21 years showed that 61% were 25-(OH)D_3_ insufficient and 9% deficient [17]. In adults, up to 40% are 25-(OH)D_3_ insufficient and 6% deficient [18,19]. A more disturbing trend is the shift in the American population towards even lower 25-(OH)D_3_ levels without a logic explanation aside from a decrease in sun exposure as the population becomes more overweight and thus engages less in outdoor activities, next to a possible better sun protection [20]. In the face of this ominous trend, the current consensus remains that population-wide screening for vitamin D deficiency is not recommended, but to limit testing to those individuals at risk of developing deficiency (hyper- and hypoparathyroidism, kidney disease, osteoporosis, etc.) [12,21,22,23]. Despite these recommendations, the importance of vitamin D is rising and parallels an increasing trend in its testing (albeit with a significant healthcare cost) [22,23].

As the aforementioned stresses the controversy about cut-off values, there is even more discussion about the correct substitution regimen. It is demonstrated that with a daily dose of up to 1000–2000 IU (25–50 µg) per day, there is a linear dose-response curve between vitamin D intake and serum 25-(OH)D_3_, which flattens at higher intakes [23]. Based on randomized controlled trials it has been calculated that an intake of 1040 IU (26 µg) per day is required in vitamin D deficiency and 400 IU (10 µg) per day in vitamin D insufficiency in order to obtain a concentration >20 ng/mL (50 nmol/L) in 97.5% of the population [24]. 

Several studies have tried to determine the optimal dosing regimens to correct deficiency. Irrespective of the interval or the exact substitution dose, most dosing regimens result in adequate serum 25-(OH) D_3_ levels, although higher doses and especially loading regimens result in a faster accomplishment of sufficiency [25,26,27,28,29,30,31,32,33,34].

Once adequate vitamin D values are reached, to further preserve adequate vitamin D levels in adults, the IOM recommends a daily dose of 600 IU per day, while the Endocrine Society recommends a dose of 600–2000 IU per day (according to the amount of sunlight the individual is exposed to) [12,35,36]. To explain the role of sunlight, it is estimated that a daily exposure of 7–30 min (depending on skin color, latitude, and season) is required to meet vitamin D substitution doses [35]. 

One of the major caveats in the use of vitamin D substitution is the risk of excessive substitution resulting in renal failure and cardiac arrest because of hypercalcemia. Both the European Food Safety Authorization and the IOM determined that the upper tolerable limit of vitamin D intake in adults is 4000 IU/day (100 µg/day) [36,37], mostly because there seems to be no additional health benefit in doses higher than 4000 IU/day [38].

## 2. Vitamin D’s Role in Immune Function: In Vitro Data

The importance of vitamin D in the regulation of both the innate and adaptive immune system was demonstrated by the discovery of the presence of VDR expression in almost all cells of the immune system, as well as the presence of the metabolizing hormones in immune cells [39,40]. Also, gut epithelial VDR is important in protecting the mucosal barrier integrity and regulating the gut innate immunity (recently demonstrated by innate lymphoid cells) [41,42,43]. The effect of vitamin D on immune cells is complex, as illustrated by the fact that VDR expression in immune cells is differently controlled according to their corresponding activation status. For example, T-cells gain a higher concentration of VDR upon activation with an increase that is already significant after eight hours and reaches a maximum 48 h after activation [44]. Monocytes on the other hand lose VDR expression by differentiating into either macrophages or dendritic cells (DCs) [45]. In immune cells, the 1α-hydroxylase enzyme, although the same enzyme as in the renal tubules, is not regulated by negative feedback by 1,25-(OH)_2_D_3_ itself [46]. As immune cells also express 24-hydroxylase, this is only minimally regulated by 1,25-(OH)_2_D_3_ and depends on the activation status of the immune cells [45,47,48,49]. Essentially, vitamin D results in a shift in the immune status towards a more tolerogenic status [4].

### 2.1. Innate

#### 2.1.1. Monocyte/Macrophage

Both monocytes as macrophages express the VDR, but as monocytes differentiate towards macrophages, there is a decrease in the expression levels of the VDR [45]. Additionally, the expression of the 1α-hydroxylase enzyme on monocytes and macrophages is upregulated by immune stimuli (i.e., signal transducer and activator of transcription-1α (STAT-1α), interferon-γ (IFN-γ), lipopolysaccharide (LPS), toll like receptors (TLR)) [50,51]. 1,25-(OH)_2_D_3_ results in an anti-inflammatory activity on macrophages as it increases interleukin (IL)-10 and decreases inflammatory stimuli (i.e., IL-1β, IL-6, tumor necrosis factor-α (TNF-α), receptor activator of nuclear factor kappa-Β ligand (RANKL), and cyclo-oxygenase-2 (COX-2)) [3]. The downregulation of inflammatory cytokines happens through the upregulation of mitogen-activated protein kinase (MAPK) phosphatase (MKP)-1 by 1,25-(OH)_2_D_3_ and subsequent inhibition of LPS-induced p38 activation [52]. 

Another pathway of inhibition of inflammatory cytokines is through inhibition of COX-2 expression by targeting thioesterase superfamily member 4 (an Akt modulator protein) [53]. In addition, activation of the TLR results in increased expression of VDR, however, TLR-mediated inflammation is controlled by 1,25-(OH)_2_D_3_ as it stimulates suppression of cytokine signaling 1 (SOCS-1) through miRNA-155 downregulation [54,55]. 1,25-(OH)_2_D_3_ has a direct antimicrobial role in monocytes and macrophages by induction of cathelicidin antimicrobial peptide (CAMP), with an increase of hCAP18 and LL-37, and by targeting defensing β2 (DEFB4) [56,57,58]. 1,25-(OH)_2_D_3_ also has an anti-oxidative effect on monocytes by upregulation of glutathione reductase (GR) and glutamate-cysteine ligase (GCL), which results in the reduced formation of oxygen radicals (such as reactive oxygen species (ROS) [59,60]. Some controversy exists in the involvement of 1,25-(OH)_2_D_3_ in the expression of inducible nitric oxide synthase (iNOS) and nitric oxide (NO) formation, as some describe upregulation, but others report inhibition [61,62]. Recent studies suggest that 1,25-(OH)_2_D_3_ is able to modulate the epigenome of immune cells, especially monocytes during antigen encounter and differentiation of the innate immune system [11,63]. 

#### 2.1.2. Dendritic Cells

**Figure 1:**
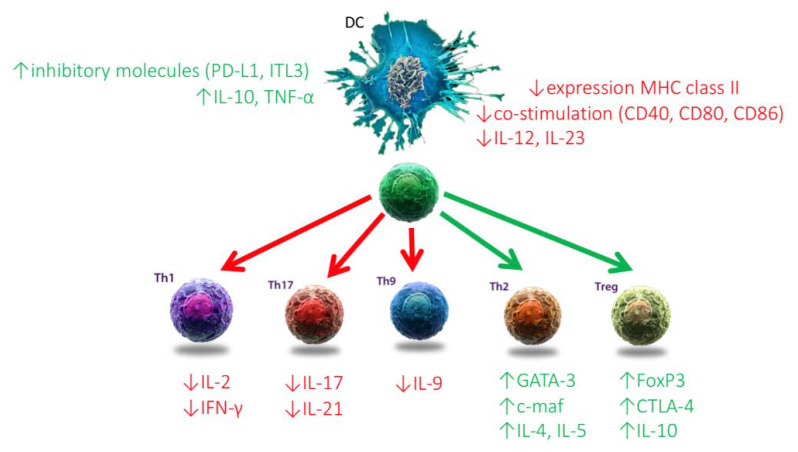
Immunomodulatory actions of active vitamin D (1,25-dihydroxyvitamin D_3_; 1,25-(OH)_2_D_3_). Both the direct as the indirect effects on T-lymphocytes are shown as 1,25-(OH)_2_D_3_ exerts its effect through direct binding on both the vitamin D receptor of the antigen-presenting cell (APC), in this case the dendritic cell (DC), and the T-lymphocytes directly. The effect of 1,25-(OH)_2_D_3_ on the APC is both an upregulation of the direct inhibition of the APC, as well as a downregulation of its antigen presentation function. The direct effect of 1,25-(OH)_2_D_3_ on the T-lymphocytes is a change towards a more tolerogenic state with an induction of Thelper-2 (Th2)-lymphocytes and regulatory T-lymphocytes (Tregs; depicted in green text), together with a downregulation of the pro-inflammatory Thelper-1 (Th1)-lymphocytes, Thelper-17 (Th17)-lymphocytes, and Thelper-9 (Th9)-lymphocytes (depicted in red text). Other abbreviations: IL: interleukin; IFN-γ: interferon-γ; TNF-α: tumor necrosis factor-α; ILT-3: immunoglobulin-like transcript-3; GATA-3: GATA binding protein-3; FoxP3: forkhead box P3, CTLA-4: cytotoxic T lymphocyte associated protein-4.

1,25-(OH)_2_D_3_ modulates DCs towards a less mature and more tolerogenic phenotype with changes in both morphology (more adherent spindle-shaped cells), as in cytokine production and surface markers [64,65,66]. There is a decreased expression of major histocompatibility complex (MHC) II, cluster of differentiation (CD) 80, CD86 (co-stimulatory molecules), and CD54 (adhesion molecule), and increased expression of CCR5 (chemokine receptor), DEC205 (antigen-uptake receptor), F4/80 (macrophage marker), and CD40 [65,67,68]. The cytokines IL-6 and IL-12 decrease together with an increase in IL-10 [3,65,67,68]. 1,25-(OH)_2_D_3_ upregulates the expression of immunoglobulin-like transcript (ILT)-3 and programmed death-ligand 1 (PD-L1), the latter contributing to the induction of regulatory T-cells (Tregs) [69]. TNF secretion by DCs appears to be another essential regulator in the induction of antigen-specific suppressive T-cells by 1,25-(OH)_2_D_3_ (Figure 1) [70].

#### 2.1.3. Others

Both natural-killer (NK) cells and neutrophils express the VDR [71]. 1,25-(OH)_2_D_3_ has an antithetical effect on neutrophils helping to minimize damage by pathogens. On the one hand, 1,25-(OH)_2_D_3_ increases the destructive power against pathogens by increased expression of cathelicidin, α and β-defensins [72,73]. Moreover, 1,25-(OH)_2_D_3_ helps to reduce bystander destruction of the pro-inflammatory response by reducing expression of Trappin-2/elafin/skin-derived anti-leucoproteinase (an inhibitor of elastase, associated with a pro-inflammatory response) and reducing migration of neutrophils [74,75,76]. Furthermore, vitamin D has been shown to reduce the formation of neutrophil extracellular traps (NETs), and thus reduces both the response against invading pathogens (NETs trap and kill pathogens) as well as the risk of autoimmunity (autoantigens are exposed and the complement system is activated in NETs) [77,78]. The effect of 1,25-(OH)_2_D_3_ on NK cells is also immunoregulatory as it results in a decreased expression of IFN-γ, CD107a (suggests decreased cytotoxic activity), and granzymes A and B [71,79,80]. 

### 2.2. Adaptive

#### 2.2.1. T-lymphocytes

1,25-(OH)_2_D_3_ can both directly and indirectly influence T-lymphocytes. The indirect pathway involves the modulation of the T-lymphocyte stimulatory function of antigen-presenting cells (APC). In monocytes and macrophages, 1,25-(OH)_2_D_3_ downregulates surface expression of MHC class II and co-stimulatory molecules (such as CD40, CD80, and CD86), and thus decreases antigen presentation [81]. 1,25-(OH)_2_D_3_ has the same effect in DCs and also inhibits their production of IL-12 and IL-23, besides a stimulation of the release of IL-10 and macrophage inflammatory protein-3α (MIP-3α) [82]. Taken together the indirect effect of 1,25-(OH)_2_D_3_ (especially on DCs as they are believed to be its central target) is a modulation of T-lymphocyte response. There will be a decrease in autoreactive T-lymphocyte proliferation, induction of both early (annexin V^+^/PI^-^) and late (annexin V^+^/PI^+^) apoptosis of autoreactive T-lymphocytes, and even a rise of Tregs [49,83]. The DC derived cytokines will alter the Thelper (Th)–lymphocyte balance from a Th1 and Th17 predominance towards a Th2 phenotype (Figure 1) [82,84]. 

The direct effect of 1,25-(OH)_2_D_3_ is variable, as it is dependent on the activation state of the T-lymphocyte as they gain a higher VDR concentration upon activation [44,85]. 1,25-(OH)_2_D_3_ inhibits the production of Th1 cytokines (i.e., IL-2, IFN-γ), Th17 cytokines (i.e., IL-17, IL-21), and Th9 cytokines (i.e., IL-9) [84,86,87,88]. The direct effects on Th2 cytokines are more controversial with a proclaimed upregulation of GATA binding protein-3 (GATA-3), c-maf, and IL-4 [85,89]. Likewise, there is an induction of IL-10-producing Tregs by binding to the Forkhead box P3 (FoxP3) promotor region, and increased expression of FoxP3 alongside cytotoxic T-lymphocyte antigen- 4 (CTLA-4) [82,86,90,91]. Because CTLA-4 expression is a key mechanism for Treg suppression, the upregulation of CTLA-4 by 1,25-(OH)_2_D_3_ suggests its more tolerogenic character (Figure 1) [86]. 

#### 2.2.2. B-lymphocytes

The presence of VDR in human B-lymphocytes with upregulation of both VDR as well as the 1α-hydroxylation enzyme, suggests a strong influence of vitamin D on B-lymphocytes [68]. It has been shown that 1,25-(OH)_2_D_3_ induces apoptosis of activated B-lymphocytes, and impedes the generation of plasma cells (by modulation of CD40 and thus NF-κB) and post-switch memory B-lymphocytes, without affecting B-lymphocyte differentiation [92,93,94]. It has been hypothesized that 1,25-(OH)_2_D_3_ may have a potential benefit in maintaining B-lymphocyte homeostasis in autoimmune diseases based on B-lymphocyte proliferation [71]. 1,25-(OH)_2_D_3_ also upregulates the production of IL-10 by B-lymphocytes, resulting in an additional regulatory effect [95]. Besides its direct role on B-lymphocyte function, 1,25-(OH)_2_D_3_ reduces the activation of T-lymphocytes by B-lymphocytes (by downregulation of CD86 expression and upregulation of CD74) [3,96].

## 3. The Impact of Vitamin D Deficiency on the Immune System: In Vitro and In Vivo Data

Epidemiological data link vitamin D deficiency to a defective functioning of the immune system with an increased risk of infections and a predisposition to autoimmune disease [97]. 

In particular, in the case of infections, associations have been described between 25-(OH)D_3_ deficiency and an increased risk for infections with mycobacterium tuberculosis and respiratory tract infections [98,99,100]. A large systematic review (of 10,933 subjects) showed that vitamin D supplementation (both D_2_ as D_3_) was protective against acute respiratory tract infections in a 25-(OH)D_3_ deficient population, especially in those receiving daily or weekly supplementation [101]. However, in children this protective effect could not be reproduced [102]. The mechanism by which vitamin D prevents respiratory tract infections is based on in vitro research that shows that 1,25-(OH)_2_D_3_ results in increased expression of cathelicidin, regulation of cytokine release, and suppression of the adaptive response by boosting the innate immune system [103]. In children and adults vitamin D_3_ as an adjunct to antibiotics did not have an additional beneficial effect in the treatment of acute bacterial pneumonia, although there was evidence that there was a trend towards faster resolution of radiographic manifestations in those with low baseline 25-(OH)D_3_ levels [104,105]. 

As vitamin D has an important effect on macrophages, and tremendous effort has been put into linking vitamin D to tuberculosis. It has been demonstrated that 25-(OH)D_3_ deficiency increases the risk of developing active tuberculosis [106]. Possible reasons are that 1,25-(OH)_2_D_3_ leads to activation and enhanced mycobactericidal activity of macrophages by induction of CAMP and DEFB4 [107,108]. Furthermore, adding vitamin D supplementation (both D_2_ as D_3_) to anti-tuberculosis treatment has been shown to have a beneficial effect [109]. Even in chronic obstructive pulmonary disease (COPD), it has been demonstrated that patients with COPD were more likely to suffer from 25-(OH)D_3_ deficiency than matched healthy smokers, with a deterioration of COPD-classification and exacerbation rate associated with a further decrease in serum 25-(OH)D_3_ [110,111]. Restoring 25-(OH)D_3_ deficiency reduces incidence of exacerbations, however only in cases of severe 25-(OH)D_3_ deficiency at baseline (at least <20 ng/mL or 50 nmol/L) [112,113].

In autoimmune diseases, there is a clear association between 25-(OH)D_3_ deficiency and the incidence of autoimmunity. In type 1 diabetes (T1D), multiple sclerosis (MS), rheumatoid arthritis (RA), systemic sclerosis (SSc), systemic lupus erythematosus (SLE), and inflammatory bowel disease (IBD), circulating levels of 25-(OH)D_3_, independent of seasonal variation and latitude, are decreased at disease onset as well as during follow-up [114,115,116,117,118,119,120,121,122,123]. A systematic review (of 5942 subjects) demonstrated that in both MS as in T1D, as principal examples of immune-mediated diseases, there was a lower level of 25-(OH)D_3_ in affected patients than in the healthy control group [124]. In SLE, lower levels of 25-(OH)D_3_ were even associated with an increased frequency of lupus flares [125]. 

This was not completely confirmed in SSc, as the diffuse type has been shown to have significantly lower levels of 25-(OH)D_3_ than the limited type; although within both types, the level per se is not associated with disease severity [119]. In general, because only a relatively small percentage of the general population has a 25-(OH)D_3_ sufficiency, only a true deficiency (25-(OH)D_3_ levels less than 12 ng/mL or 30 nmol/L) seems to be correlated with an increased prevalence and aggressiveness of autoimmune diseases [124,126]. 

The link between vitamin D and autoimmune disease is furthermore supported by both seasonal variation (increased prevalence in children born in spring) and latitude (higher prevalence in northern countries with less UVB radiation) [127,128,129,130,131,132]. Additional evidence for the link between vitamin D and autoimmunity is found on the genome level as single nucleotide polymorphisms (SNPs) in three key vitamin D metabolism genes (i.e., DHCR7 and CYP2R1: determinants of circulating 25-(OH)D_3,_ and CYP27B1: vitamin D signaling in T-lymphocytes) and VDR genes (i.e., TaqI and BsmI) have been linked to an increased risk of respectively T1D and MS [116,133,134]. Furthermore, SNPs in VDR genes (i.e., ApaI, BgII GT and TaqI) are associated with SSc and IBD, respectively [135,136]. Since these SNPs result in a decreased efficacy of vitamin D substitution, this supports the importance of vitamin D on autoimmunity. Moreover, there is an association between certain VDR polymorphisms and autoimmunity as VDR FokI and TaqI polymorphisms are associated with an increased risk in SLE and RA [137,138]. 

As the importance of restoring a 25-(OH)D_3_ deficiency was stressed by its role in infections as well as in immune-mediated diseases, this was extrapolated to critically ill patients, where a distinct association was demonstrated between low 25-(OH)D_3_ levels and adverse outcomes (both in morbidity as in mortality) [139,140,141,142,143,144,145]. It has been shown that in critically ill patients 25-(OH)D_3_ even continue to decrease if no substitution is started [140]. In contrast to these findings in adults, a recent large meta-analysis in critically ill children was not able to link a 25-(OH)D_3_ deficiency to a higher mortality [146]. The reason for a lower 25-(OH)D_3_ in critically ill patients (both at admission and the subsequent decrease) is still not completely understood. One explanation is a decrease in DBP due to reduced protein synthesis and increased clearance (although reduced levels of DBP do not affect the free 25-(OH)D_3_ concentration) [140]. Another possible explanation is that low 25-(OH)D_3_ is a marker of illness as inflammatory processes reduce 25-(OH)D_3_, so an important consideration in critically ill patients might be that serum 25-(OH)D_3_ is the consequence of illness and not the cause [147,148].

The exact mechanism of protection by vitamin D_3_ remains elusive. Based on current knowledge, the most important observation is that vitamin D_3_ results in a shift from an inflammatory Th1 response towards a pro-tolerogenic Th2 response with an arrest of cytotoxic T-lymphocyte infiltration and an increase in CD4^+^CD25^+^ Tregs [149,150,151]. In healthy individuals, vitamin D_3_ increases the absolute number of Tregs without altering their suppressor function [152]. On the other hand, a similar dose of vitamin D_3_, in vivo, was shown to improve the suppressor function of Tregs, in T1D and MS patients, without altering their absolute number [153,154,155,156,157]. Of note, the baseline frequency and absolute number of Tregs in peripheral blood before initiation of vitamin D_3_ supplementation was not different between healthy controls and patients [158]. It is clear that the exact effect on Tregs is still not completely known, as in SLE (in vivo), vitamin D_3_ resulted in an increase in Tregs, independent of the patients’ vitamin D status [159]. Besides its major impact on Tregs, vitamin D_3_ is able to directly reduce effector T-lymphocytes as has been demonstrated, in vivo, in both MS as SLE [160,161].

The change towards a more tolerogenic status is also reflected by a change in the cytokine profile. In vitro, 1,25-(OH)_2_D_3_ resulted in a reduction of IL-1, IL-6, and IL-17, together with a reduction in TNF-α, as demonstrated in RA, and an increase in IL-4, IL-5, and IL-10 and a reduction in IFN-γ, as demonstrated in IBD [162,163,164,165,166]. To extrapolate these in vitro findings to in vivo studies is more complicated, however results are also pointing towards a more tolerogenic state, as in MS there was a decrease in IL-17 and an increase in IL-10 with the latter also being observed in IBD [167,168,169,170]. Furthermore, in vivo, there was a decrease in inflammatory cytokines IL-23 and IL-17 in RA and even a direct antifibrotic effect by impairment of TGF-β in SSc [171,172].

Furthermore, 1,25-(OH)_2_D_3_ and even 25-(OH)D_3_ affect the maturation and migration of DCs, conferring an immunoregulatory role and tolerogenic phenotype, characterized by IL-10 production and thus again promoting tolerance [173,174]. This finding is supported by the comparison of transcriptomes of 1,25-(OH)_2_D_3_/dexamethasone-modulated tolerogenic with non-modulated mature inflammatory DCs, and it was shown that these modulated DCs had immunomodulating effects, including the induction of Tregs [175,176]. In addition, in SLE, both in vitro and in vivo experiments show that vitamin D counterbalances B-lymphocyte hyperactivity by inducing early apoptosis in B-lymphocytes with a possible favorable effect [94,161,171].

## 4. Vitamin D Metabolites as Immune Modulators in Autoimmune Diseases: Animal Models and Human Data

As vitamin D is an important regulator of the immune system with a preponderance towards tolerance induction, its therapeutic potential as an immune modulator is appealing in the treatment of immune-mediated diseases. Studies in animal models of autoimmune diseases show that restoring serum 25-(OH)D_3_ levels using high doses of vitamin D metabolites (i.e., 25-(OH)D_3_ and 1,25-(OH)_2_D_3_) or less calcemic vitamin D analogues, can alter the course of autoimmune diseases like T1D, MS, or RA [177]. One of the first studies to show a decrease in T1D incidence by vitamin D was performed in the non-obese diabetic (NOD) mouse model, which was able to demonstrate a significant decrease in T1D incidence due to long-term treatment with high dose 1,25-(OH)_2_D_3_ starting from a young age [151,178,179]. Later in this model it was demonstrated that T1D could be arrested by treatment with a 1,25-(OH)_2_D_3_ analog, possibly by increasing Tregs and inhibition of Th1-lymphocytes [150,180]. In the experimental autoimmune encephalitis (EAE) mouse model of MS, the effect on disease alterations has been even more intensely studied, with an inhibition of EAE by increasing IL-4 and transforming growth factor-β1 (TGF-β1) and by modulation of the JAK-STAT pathway in the IL-12/IFN-γ axis [181]. In the pristane-induced model of SLE, there was only a reduction in IFN-γ, but no effect on IL-4 [182]. In addition, a reduction in the pro-inflammatory cytokines IL-1β, IL-6, IL-8 and prostaglandin E2 was described in the type II collagen injection rat model of RA [183]. Recently in the Act1-/- mouse model of SLE and Sjögren syndrome, amongst other changes characterized by a peripheral B-lymphocyte expansion, it was demonstrated that lower levels of vitamin D_3_ are linked to an increase in memory B-lymphocytes [184]. Furthermore, in collagen-induced arthritis, the mouse model of RA 1,25-(OH)_2_D_3_ was able to decrease severity of arthritis by downregulation of Th17 cells and increasing Tregs [185]. 

Later these animal models were translated to humans as the European Diabetes Centers EURODIAB study showed a significant decrease in T1D onset in children receiving vitamin D supplementation [186]. The first major birth-cohort study was done in Finland and resulted in a staggering 80% reduction in T1D onset in 10,336 children receiving vitamin D supplementation before the age of 1 year, irrespective of the dose of supplementation [187]. Likewise, in MS, low neonatal 25-(OH)D_3_ concentrations are associated with an increased risk of disease incidence [126]. Based on cohort data of more than 7 million persons, it was proven in MS that vitamin D supplementation resulted in significant lower disease incidence [188,189]. 

As it was demonstrated in both animal and human studies that the age of intervention was critical in disease prevention, the rationale was that ensuring adequate 25-(OH)D_3_ levels as early as during pregnancy would be critical for disease prevention. In MS, a large systematic review showed that 25-(OH)D_3_ deficiency during pregnancy resulted in an increased prevalence of MS in the offspring [190]. Therefore, in theory, maternal vitamin D supplementation during pregnancy would seem protective. However, in T1D, studies indicated that maternal vitamin D supplementation during pregnancy did not reduce the risk on T1D in the offspring [191,192,193]. Even a recent large cohort study, The Environmental Determinants of Diabetes in the Young (TEDDY), conducted on 8676 European and American children with T1D-associated human leukocyte antigen (HLA) genotypes, and thus an increased risk for the development of islet autoimmunity and T1D, was not able to demonstrate an effect of maternal vitamin D supplementation on islet-autoimmunity in the offspring [194]. Unfortunately, to our knowledge there are also no studies investigating the effect of vitamin D supplementation during pregnancy on the incidence of MS or rheumatic diseases in offspring. An important note in a lot of these (smaller) studies, and one of the main reasons for continuing debate, is that most of these studies are performed in retrospect resulting in inconsistency in timing of the start of intervention, difference in dosing regimens and eventually conflicting results [193,195,196,197,198,199,200]. It is because of these conflicting results that the aim is currently changing into identifying subpopulations predicted to benefit most from vitamin D supplementation. The earlier mentioned TEDDY cohort study, conducted in children with T1D-associated HLA genotypes (and thus an increased risk for the development of islet autoimmunity and T1D), demonstrated that vitamin D supplementation seemed to be only beneficial in those with minor alleles at VDR ApaI [201]. Others found similar results in those homozygous for the VDR Cdx2 G/G [202]. These studies are examples of future tailored medicine as specific VDR genotypes seem to have distinct functional effects on immune cells, e.g., depending on the Fokl polymorphism, the effect of both 25-(OH)D_3_ and 1,25-(OH)_2_D_3_ results in different functional effects on lymphocytes and monocytes [203,204].

## 5. Discrepancy between Promising in Vitro Data, Animal Models and Human Intervention Trials

Whereas the hypothesis that vitamin D and its metabolites have a role in normal physiology as immune modulators is now well supported by in vitro studies showing that there is a dose-dependent effect of vitamin D or its metabolites and even synthetic analogues on many immune cell subsets, the translation of these observations into solid results in clinical trials has failed and the scientific community is starting to question the relevance of the in vitro observations and even interventions in animal models for human health. So, what is missing? Why are the in vitro observations not translated in success in clinical intervention trials? 

### 5.1. Dose of Vitamin D Products

Studying effects of vitamin D_3_ (i.e., metabolites or analogues) on immune cells in vitro is an artificial situation, with continued exposure of these isolated immune cell subsets and too high doses of vitamin D products, mostly 1,25-(OH)D_2_D_3_, being induced. Often supra-physiological concentrations are employed that are not achievable in human peripheral blood. Still, these concentrations could be achieved in local sites of inflammation, as also many immune cells can produce vitamin D products themselves upon activation [68,82]. Thus, the physiological relevance of the in vitro observations seems valid, but the translation to interventions with supplements of vitamin D products is problematic. 

In animal models, it has been shown that in order to see any effect on disease modulation, the dose and route of administration of the vitamin D products are crucial. As such, in the T1D studies in NOD mice, therapy was only successful when doses of vitamin D or its metabolites or analogues were used that were at the edge of toxicity [178,180,205]. In addition, continuous administration, leading to continued exposure to the high doses of the products was needed, often lifelong [180]. 

When looking at human intervention trials, many used safe doses of regular vitamin D, lifting levels of 25-(OH)D_3_ above sufficiency levels, but far from the very high levels observed in those animal studies that measured levels and correlated them to efficacy of treatment. In NOD mice, for example, we showed that a very high dose of regular vitamin D was able to prevent T1D, but this study was also done using a very high dosing regimen that for humans would require a lifelong daily dose of 12,500 IU [180]. 

Translating in vitro studies and animal studies to humans, would mean using much higher doses of vitamin D products and thus reaching levels at which also side effects of hypercalcemia would be seen [205,206,207]. In many studies in animals, investigators avoided hypercalcemia by lowering calcium intake of the animals, a detail often not noted by readers of the manuscripts [208].

In addition, in the in vitro experiments, continued exposure of immune cells to the vitamin D products was used, and in most animal models treatment was given continuously (via food supplementation or oral gavage). In human studies, often intermittent administration or bolus doses are used to improve compliance. These bolus doses have rarely been tested in animal models and have the potential to induce completely different immune effects than the continuous exposure used in vitro and in animal studies. Indeed, exposure to extremely high doses of vitamin D products of immune cells in vitro induces very different effects, such as apoptosis or even necrosis of specific cell types [209,210]. Also in animals, a bolus injection of vitamin D induces even the opposite effect for instance on macrophages, paralyzing them, rather than making them more efficacious [211]. In those clinical studies where continued administration of vitamin D products was studied, there is the major issue of compliance, which is not an issue in in vitro studies or animal studies. 

Finally, the immune effects of vitamin D products in vitro are not a full immune suppression, but rather an immune modulation, shifting the adaptive immune system towards tolerance to antigens and the innate immune system to a better viral and bacterial clearance (as discussed above). These effects are subtle and may not be sufficient to achieve dramatic effects by themselves when administered in monotherapy in human disease. So, rather than studying the effect of vitamin D products alone, combinations may be needed. 

### 5.2. Timing of Intervention and Duration of Exposure

In vitro studies typically start with naïve immune cells, freshly isolated from immune organs or blood of healthy subjects, which are then exposed for days to vitamin D_3_ products. In animal models, likewise, therapy is often started before disease onset (most autoimmune models) or early on in the disease. Few studies have looked at effects once disease is overt, and those that have indicate that at that stage vitamin D products by themselves, even at high doses, are not disease altering anymore [212]. In humans however, most intervention studies are late stage (disease is present) and study vitamin D monotherapies. In addition, in animal models, therapy is maintained for weeks and months, often during the whole life of the animal, whereas in humans, shorter duration studies happen.

### 5.3. Relevance of Animal Models for Human Disease

A major weakness in translation of our observations in vitro and confirmatory studies in animal models to humans, is the relevance of the animal models studied. In autoimmune diseases, animal models are criticized as they are, for instance, induced (most animal models of EAE and RA), using antigens that are sometimes not relevant for human disease, or have a much more dramatic course (e.g., NOD T1D mouse model) than what is observed in humans. 

Thus, success in these animal models, using high doses of vitamin D products, starting therapy before induction or before disease is present, treating animals for long periods is not necessarily a guarantee for success in human disease. 

## 6. Concluding Remarks

There is an indisputable relation between vitamin D and the immune system. With respect to in vitro, overwhelming evidence exists for a physiological role for the vitamin D system in immune regulation, and immune modulation can be observed by exposing immune cells to pharmacological doses of vitamin D metabolites. In animal models and humans, a correlation exists between adverse immune outcomes (infections and autoimmune diseases) and vitamin D deficiency, but translation of the in vitro observations of active vitamin D_3_ on the immune system to solid results of regular vitamin D supplementation in clinical trials have mostly failed. An important reason might be that the choice of the vitamin D metabolite, as well as its dose and frequency of administration are critical factors that need to be considered when designing clinical trials. Many in vitro effects on isolated immune cells are induced by supra-physiological concentrations of 1,25-(OH)D_2_D_3_, which are probably not achievable with regular vitamin D supplements in humans, as these concentrations risk hypercalcemia and soft tissue calcifications. Moreover, recurrent use of regular vitamin D, for instance daily or weekly (in comparable cumulative doses) instead of every 6–12 months, may enhance long-term compliance depending on the lifestyles of the target groups. In addition, the timing of vitamin D intervention will be crucial. In animal models, vitamin D metabolites work best in a preventive setting, a time window that is often missed in human trials. Therefore, future randomized and controlled trials will be needed to investigate whether supplementation with regular vitamin D can indeed prevent or modify the course of inflammatory or autoimmune diseases in at-risk subjects. For now, the bottom line on the effect of vitamin D in the immune system is that avoidance of severe vitamin D deficiency improves immune health and decreases susceptibility to autoimmune diseases.
